# Designing small universal *k*-mer hitting sets for improved analysis of high-throughput sequencing

**DOI:** 10.1371/journal.pcbi.1005777

**Published:** 2017-10-02

**Authors:** Yaron Orenstein, David Pellow, Guillaume Marçais, Ron Shamir, Carl Kingsford

**Affiliations:** 1 Computer Science and Artificial Intelligence Laboratory, MIT, Cambridge, Massasschusetts, United States of America; 2 Blavatnik School of Computer Science, Tel-Aviv University, Tel-Aviv, Israel; 3 Computational Biology Department, School of Computer Science, Carnegie Mellon University, Pittsburgh, Pennsylvania, United States of America; Princeton University, UNITED STATES

## Abstract

With the rapidly increasing volume of deep sequencing data, more efficient algorithms and data structures are needed. Minimizers are a central recent paradigm that has improved various sequence analysis tasks, including hashing for faster read overlap detection, sparse suffix arrays for creating smaller indexes, and Bloom filters for speeding up sequence search. Here, we propose an alternative paradigm that can lead to substantial further improvement in these and other tasks. For integers *k* and *L* > *k*, we say that a set of *k*-mers is a *universal hitting set* (UHS) if every possible *L*-long sequence must contain a *k*-mer from the set. We develop a heuristic called DOCKS to find a compact UHS, which works in two phases: The first phase is solved optimally, and for the second we propose several efficient heuristics, trading set size for speed and memory. The use of heuristics is motivated by showing the NP-hardness of a closely related problem. We show that DOCKS works well in practice and produces UHSs that are very close to a theoretical lower bound. We present results for various values of *k* and *L* and by applying them to real genomes show that UHSs indeed improve over minimizers. In particular, DOCKS uses less than 30% of the 10-mers needed to span the human genome compared to minimizers. The software and computed UHSs are freely available at github.com/Shamir-Lab/DOCKS/ and acgt.cs.tau.ac.il/docks/, respectively.

## Author summary

High-throughput sequencing data has been accumulating at an extreme pace. The need to efficiently analyze and process it has become a critical challenge of the field. Many of the data structures and algorithms for this task rely on *k*-mer sets (DNA words of length *k*) to represent the sequences in a dataset. The runtime and memory usage of these highly depend on the size of the *k*-mer sets used. Thus, a minimum-size *k*-mer hitting set, namely, a set of *k*-mers that hit (have non-empty overlap with) all sequences, is desirable. In this work, we create universal *k*-mer hitting sets that hit any *L*-long sequence. We present several heuristic approaches for constructing such small sets; the approaches vary in the trade-off between the size of the produced set and runtime and memory usage. We show the benefit in practice of using the produced universal *k*-mer hitting sets compared to minimizers and randomly created hitting sets on the human genome.

## Introduction

The pace of high-throughput sequencing keeps accelerating as it becomes cheaper and faster and with it the need for faster and more memory efficient genomic analysis methods grows. The NIH Sequence Read Archive, for example, currently contains over 12 petabases of sequence data and is growing at a fast pace. Increased use of sequence-based assays (DNA sequencing, RNA-seq, numerous other “*-seq”s) in research and in clinical settings creates high computational processing burdens. Metagenomic studies generate even larger sequencing datasets. New fundamental computational ideas are essential to manage and analyze these data.

The minimizer approach has been extremely successful in increasing the efficiency of several sequence analysis challenges. Given a sequence of length *L*, its *minimizer* is the lexicographically smallest *k*-mer in it [1, 2]. For a sequence *S* of any length its *minimizer set* is the set of minimizers of every *L*-long subsequence in *S*. Hence, every window of length *L* in *S* is represented in the set, and a minimizer set for a sequence *S* constitutes a succinct representation for it. As we discuss below, minimizers have had numerous applications in sequence analysis.

Here, we generalize and improve on the minimizer idea. To avoid dependence on a particular sequence *S*, we introduce the notion of a universal hitting set. For integers *k*, *L*, a set *U*_*k*,*L*_ is called a *universal hitting set* of *k*-mers (UHS) if every possible sequence of length *L* must contain at least one *k*-mer from *U*_*k*,*L*_. The set of all *k*-mers is a trivial UHS, but it does not provide any useful reduction in computational resources needed. Hence, our main computational problem is:

*Problem 1*. Given *k* and *L*, find a smallest universal hitting set of *k*-mers.

A small UHS has a variety of applications in speeding up genomic analyses since it can be used where minimizers have been used in the past. For example:

1. **Hashing for read overlapping.** A naïve read overlapper must test *O*(*n*^2^) pairs of reads to see whether they overlap (where *n* is the number of reads). If we require an overlap of length *L*, any pair of reads with such an overlap must share a *k*-mer from set *U*_*k*,*L*_ in this overlapped region. By bucketing reads into bins according to the universal *k*-mers they contain, we need only test pairs of reads in the same bucket. The number of buckets is limited by |*U*_*k*,*L*_|.
2. **Sparse suffix arrays.** A sparse suffix array of a string *S* saves memory by storing an index for only every *s*th position in *S* [3]. To query a sparse suffix array for string *q*, we perform at most *s* queries starting from indices 0, …, *s* − 1 in *q*; one of these queries will intersect a position stored in the suffix array. Using *U*_*k*,*L*_, we can instead store only positions in *S* that start with a *k*-mer in *U*_*k*,*L*_. Any query with |*q*| ≥ *L* must contain one of these selected *k*-mers and will be matched when searching the suffix array. This approach has been applied with minimizers [4] to good effect.
3. **Bloom filters to speed up sequence search.** Bloom filters have been used to speed up sequence search by storing *k*-mers present in a read set for quick testing [5, 6]. In current implementations, all *k*-mers present in a read set are stored in these filters. If, instead, only the set of *k*-mers in *U*_*k*,*L*_ is stored, any window of length ≥ *L* is still guaranteed to contain one of these representative queries, potentially reducing the size of Bloom filters that must be maintained.

Minimizers have been used for some of these and similar applications [4, 7–9]. They were originally introduced by Roberts *et al*. [1] for genome assembly. The same idea was introduced independently for plagiarism detection in Schleimer *et al*. [2]. For example, MSP [10] compresses *k*-mers by hashing them to their 4-mer minimizer to efficiently construct a de Bruijn graph for assembly. SparseAssembler [11] represents the de Bruijn graph using only every *g*-th *k*-mer in the sequence (and has also been implemented using minimizers). Kraken [12] uses minimizers to speed up database queries for *k*-mers during metagenome sequence classification. KMC 2 [8] uses minimizers to cluster subsequences for counting *k*-mer occurrences. The Locally Consistent Parsing (LCP) [13] algorithm provides the concept of “core substrings” which, like minimizers, are guaranteed to be shared by long enough identical strings. SCALCE [14] uses core substrings to compress DNA sequences.

A small UHS, if it can be found, has a number of advantages over minimizers for these applications:

1. The set of minimizers for a given collection of reads may be as dense as the complete set of *k*-mers (size |Σ|^*k*^ for an alphabet Σ), whereas we show that we can often generate UHSs smaller by a factor of nearly *k*. We also demonstrate on real genomic sequences that the number of UHS *k*-mers needed to process them is substantially smaller.
2. For any *k* and *L*, a set of universal *k*-mers needs to be computed only once and not recomputed for every dataset.
3. The hash buckets, sparse suffix arrays, and Bloom filters created for different datasets will contain a comparable set of *k*-mers if they are sampled according to a UHS. This will enable easier comparison and integration of the datasets.
4. One does not need to look at the reads or to build a dataset-specific de Bruijn graph in order to decide which *k*-mers to use.

Problem 1 can be rephrased as a problem on the complete de Bruijn graph of order *k* (see Definition 1 below). This is the viewpoint we take for most of this study:

*Problem 2*. Given a de Bruijn graph *D*_*k*_ of order *k* and an integer *L*, find a smallest set of vertices *U*_*k*,*L*_ such that any path in *D*_*k*_ of length ℓ = *L* − *k* passes through at least one vertex of *U*_*k*,*L*_.

Here and throughout, the length of a path is the number of *edges* in it. We show that the related problem of finding a minimum-size *k*-mer set that hits every string in a given set $\tilde{S}$ of *L*-long strings is NP-hard. This problem differs from ours, in that the set $\tilde{S}$ is part of the input. However, the fact that finding a small set of *k*-mers that hits every sequence in a particular data set is hard further motivates the need for a universal set that can be computed once for any input sequence. Our main contribution is an algorithm called DOCKS that finds a compact set of *k*-mers that hits any *L*-long sequence. We also provide several variants of the algorithm, trading-off some solution quality for speed. We show empirically that the produced sets are often close to a theoretical lower bound, implying their near-optimality. Our use of a greedy heuristic is motivated by the fact that finding a minimum-size ℓ-long path cover in a graph *G* is NP-hard when *G* is a directed acyclic graph (DAG). We report on the size of the universal *k*-mer hitting set produced by DOCKS and demonstrate on genomic datasets that we can more uniformly cover sequences with a smaller set of *k*-mers than is possible using minimizers. For example, we show that the number of *k*-mers needed to cover the human genome using a UHS is less than one third of that required by minimizers.

The software to compute small UHSs is freely available at github.com/Shamir-Lab/DOCKS/. Universal sets of *k*-mers computed by DOCKS for a range of values of *L* and *k* are freely available at acgt.cs.tau.ac.il/docks/. A preliminary version of this study appeared in [15].

### Preliminaries

Throughout this paper, *k* denotes the length of a *k*-mer word, while *L* denotes the length of the long sequences.

**Definition 1 (de Bruijn Graph).** A *de Bruijn graph* of order *k* over alphabet Σ is a directed graph in which every vertex has an associated label (a string over Σ) of length *k* (*k*-mer) and every edge has an associated label of length *k* + 1. There are exactly |Σ|^*k*^ vertices in a de Bruijn graph, each representing a unique *k*-mer. If an edge (*u*, *v*) has label *l*, then the label of *u* must be the *k*-prefix (prefix of length *k*) of *l* and the label of *v* must be the *k*-suffix (suffix of length *k*) of *l*. A *complete* de Bruijn graph contains all possible edges of this type, which represent together all (*k* + 1)-mers over Σ.

Every path in a de Bruijn graph represents a sequence. A path *v*_0_, *e*_0_, *v*_1_, *e*_1_, *v*_2_, …, *v*_*n*_ of length *n* spells a sequence *s* of length *n* + *k* such that the label of *v*_*i*_ occurs in *s* starting at position *i* for all 0 ≤ *i* ≤ *n*, and the label of *e*_*i*_ occurs in *s* starting at position *i* for all 0 ≤ *i* ≤ *n* − 1. Note that vertices and edges may repeat in a path.

We define terminology for *k*-mers intersecting sequences over an alphabet Σ:

**Definition 2 (hits).** We say that *k*-mer *w* *hits* string *S*, denoted *w* ⊆ *S*, if *w* appears as a contiguous substring in *S*. *k*-mer set *X* *hits* string *S* if there exists *w* ∈ *X* s.t. *w* ⊆ *S*. Define *hit*(*w*, *L*) = {*S* ∈ Σ^*L*^ ∣ *w* ⊆ *S*} for *k*-mer *w* and length *L*, where Σ^*L*^ is the set of all *L*-long substrings over alphabet Σ. Define $hit\left(X,L\right)=\underset{w\in X}{\cup }hit\left(w,L\right)$.

The universal set of hitting *k*-mers from Problem 1 is then a set *U*_*k*,*L*_ which satisfies *hit*(*U*_*k*,*L*_, *L*) = Σ^*L*^.

## Materials and methods

It is not known how to efficiently find a minimum universal (*k*, *L*)-hitting set. As we prove in the Appendix, the problem of finding a minimum (non-universal) *k*-mer set that hits a given set of input sequences is NP-hard (see Appendix, Subsection NP-hardness of MINIMUM (*k*, *L*)-HITTING SET in S1 Text). In the face of the hardness result for this related problem, we give below a practical heuristic to find a compact (near-optimal) universal *k*-mer set. This algorithm works on the de Bruijn graph of order *k* in two steps: first it finds and removes a minimum-size *k*-mer set hitting all infinite sequences, and then it finds and removes additional *k*-mers in order to hit all remaining *L*-long sequences. We now describe these two steps in detail.

### Finding a minimum *k*-mer set hitting all infinite sequences

The problem of finding a minimum-size *k*-mer set hitting all infinite sequences is known in the literature as finding an unavoidable set of constant length [16]. Note that finite words may avoid the set. Finding a minimum-size unavoidable set for a given *k* can be solved in time polynomial in the output size [16]. The original algorithm is due to Mykkeltveit [17]. Its running time is *O*(*kM*(*k*)), where *M*(*k*) is the size of the minimum unavoidable set. *M*(*k*) converges to |Σ|^*k*^/*k* (an exact formula is given in Eq 11), so the running time is *O*(|Σ|^*k*^).

An unavoidable set of constant length *k* is equivalent to a set of vertices in a complete de Bruijn graph of order *k* whose removal turns it into a directed acyclic graph (DAG). Each *k*-mer in the set corresponds to a vertex, and the removal of vertices from every cycle guarantees that no infinite sequence is represented as a path in the graph. This set is known as a *decycling set*.

### Hitting remaining length *L* sequences

Unfortunately, finding an unavoidable set is not enough, as there may be *L*-long sequences that avoid that set. Thus, we need additional *k*-mers to hit those. If we consider the graph formulation, after removal of a decycling set from the graph we are left with a DAG, which may contain (*L* − *k*)-long paths representing *L*-long sequences. We need to remove additional vertices, so that there is no path of length ℓ = *L* − *k*. The problem of finding a minimum-size set of vertices that hit all ℓ-long paths in a general directed acyclic graph is known to be NP-hard, as we review in the Appendix (see Appendix, Subsection NP-hardness of MINIMUM ℓ-PATH COVER IN A DAG in S1 Text). Therefore, we give a heuristic solution.

Our initial algorithm is based on the greedy algorithm for the minimum hitting set [18]. We define the *hitting number* *T*(*v*, *ℓ*) of a vertex *v* to be the number of paths of length *ℓ* that contain *v*. The main observation is that we can calculate the hitting number of each vertex efficiently using dynamic programming. The solution is based on calculating the number of paths of length *i* that terminate at vertex *v*, and the number of paths of length *i* that start at vertex *v*, for all *v* ∈ *V* and 0 ≤ *i* ≤ *ℓ*. Then, the number of *ℓ*-long paths through *v* is directly computable from these values by breaking any path into an *i*-long path ending at *v* and an (*ℓ* − *i*)-long path starting at *v*, for all possible values of *i*. We set *ℓ* = *L* − *k* to get the desired hitting number of each vertex.

Specifically, let *G*′ = (*V*′, *E*′) be the directed acyclic graph, after removal of the decycling set. Denote by *D* and *F* matrices of size |*V*′| × (*ℓ* + 1) where *D*(*v*, *i*) is the number of *i*-long paths in *G*′ starting at vertex *v* and *F*(*v*, *i*) is the number of *i*-long paths ending at vertex *v*.

The calculation of *D* and *F* is done recursively as follows:

$$D(v,0)=F\left(v,0\right)=1,\text{for}\;\text{all}\;v\in V^{\prime }$$ (1)

$$D(v,i)=\sum_{\left(v,u\right)\in E^{\prime }}D(u,i-1)$$ (2)

$$F(v,i)=\sum_{\left(u,v\right)\in E^{\prime }}F(u,i-1)$$ (3)

To get the number of *ℓ*-long paths that vertex *v* participates in, we sum:

$$T\left(v,\ell \right)=\sum_{i=0}^{\ell }F\left(v,i\right)\cdot D\left(v,\ell -i\right)$$ (4)

The running time is proportional to the sum of all vertex degrees (which is Θ(|*E*′|)) times *ℓ*, giving a running time of *O*(|Σ|^*k*+1^ ⋅ *ℓ*) for *ℓ* = *L* − *k*.

### The DOCKS algorithm

The full algorithm combines the two steps. First, we find a decycling set in a complete de Bruijn graph of order *k* and remove it from the graph, obtaining a DAG. Then, we repeatedly remove a vertex *v* with the largest hitting number *T*(*v*, *ℓ*) until there are no *ℓ*-long paths, recomputing *T*(*u*, *ℓ*) for all remaining vertices *u* after each removal. This is summarized below (Algorithm 1).

**Algorithm 1 DOCKS: Find a compact *k*-mer set hitting all *L*-long sequences**

1: Generate a complete de Bruijn graph *G* of order *k*, set *ℓ* = *L* − *k*.

2: Find a decycling vertex set *X* using Mykkeltveit’s algorithm.

3: Remove all vertices in *X* from graph *G*, resulting in *G*′.

4: **while** there are still paths of length *ℓ* **do**

5:  Calculate *D*(*v*, *i*) and *F*(*v*, *i*) for each vertex *v* and 0 ≤ *i* ≤ *ℓ*.

6:  Calculate *T*(*v*, *ℓ*) for each vertex *v*.

7:  Remove a vertex with maximum hitting number from *G*′, and add it to set *X*.

8: **end while**

9: Output set *X*.

Finding the decycling set takes *O*(|Σ|^*k*^). In the second phase, each iteration calculates the hitting number of all vertices in time *O*(|Σ|^*k*+1^*ℓ*). The number of iterations is 1 + *p*, where *p* is the number of vertices removed. Thus, the total running time is dominated by steps 4–8 and is *O*((1 + *p*)|Σ|^*k*+1^*ℓ*).

The exponential dependence of DOCKS on *k* limits the range of *k* to which it can be applied (see Results, Subsection DOCKS). This motivates us to develop two variants that trade larger solution sizes for faster running times in the different heuristics described next.

### The DOCKSany algorithms

In order to extend the range of *k*, *L* values beyond what DOCKS can compute in reasonable times, we develop a faster heuristic that may produce cruder solutions. Instead of calculating the number of *ℓ*-long paths through each vertex, we consider *all* paths through each vertex. This number, denoted by *T*(*v*), can be calculated more quickly and serve as an estimate of *T*(*v*, *ℓ*). We call this heuristic DOCKSany (Algorithm 2).

DOCKSany has the same structure as DOCKS, but with one difference: it removes a node *v* with maximum *T*(*v*) in each iteration. To compute *T*(*v*) for all *v*, the vertices in the current graph *G*′ = (*V*′, *E*′) are first sorted in topological order *v*_1_ ≤ … ≤ *v*_*n*_. Define *F*(*v*) as the number of paths ending at *v*. The vertices are visited in topological order and the incoming edges into *v* are used to compute:

$$F(v)=1+\sum_{\left(u,v\right)\in E^{\prime }}F(u)$$ (5)

Similarly, *D*(*v*), the number of paths starting at *v* is computed by visiting the vertices in reverse topological order and computing.

$$D(v)=1+\sum_{\left(v,u\right)\in E^{\prime }}D(u)$$ (6)

*T*(*v*) is then calculated for all vertices as:

T(v)=F(v)·D(v). (7)

A vertex *v* with maximum *T*(*v*) is removed, *G*′ is updated, and the process is repeated until there are no paths of length *ℓ* in the graph.

**Algorithm 2 DOCKSany: A faster heuristic for a compact *k*-mer set hitting all *L*-long sequences**

1: Generate a complete de Bruijn graph *G* of order *k*, set *ℓ* = *L* − *k*.

2: Find a decycling vertex set *X* using Mykkeltveit’s algorithm.

3: Remove all vertices in *X* from graph *G*, resulting in *G*′.

4: **while** there are still paths of length *ℓ* **do**

5:  Calculate *D*(*v*) and *F*(*v*) at each vertex *v*.

6:  Calculate the number *T*(*v*) of paths passing through each vertex *v*.

7:  Remove a vertex *v* with maximum *T*(*v*) from *G*′, and add it to set *X*.

8: **end while**

9: Output set *X*.

Computing *D*(*v*) for all *v* requires visiting each edge in the graph once, and hence takes *O*(|Σ|^*k*+1^). The time for computing *F*(*v*) for all *v* is the same. Hence, *T* is computable in *O*(|Σ|^*k*+1^) time. Computing the longest path in a DAG (step 4) also requires *O*(|Σ|^*k*+1^). If *p* vertices are removed, then the total runtime for this algorithm is *O*((1 + *p*)|Σ|^*k*+1^), a factor of Θ(*ℓ*) faster than the DOCKS algorithm. The space complexity is also smaller, *O*(|Σ|^*k*+1^) vs. *O*(*ℓ*|Σ|^*k*+1^) for DOCKS.

In addition to shorter runtimes and decreased memory usage, this heuristic offers one more advantage over the original DOCKS algorithm. The vertex removal choice is independent of *L*. The value of *L* only determines when the algorithm terminates. Thus, hitting sets for all values of *L* or larger can be computed in one run. This is in contrast with DOCKS, in which the hitting number of each vertex depends on *L*, and so DOCKS must be run for each desired value of *L*.

Finally, in order to calculate the hitting set for even larger *k*, we can further speed up DOCKSany as follows. In the DOCKSanyX heuristic, the top *X* vertices, ranked by the hitting number *T*(*v*), are removed (in step 7) in each iteration. This can shorten the running time of each iteration by a factor of *X*, but may produce larger hitting set solutions.

### An integer linear programming (ILP) formulation

To investigate whether optimal solutions can be found practically, we formulate the problem of the minimal universal *k*-mer hitting set as an integer linear program (ILP). In the ILP formulation there are |Σ|^*k*^ binary variables *x*_*i*_ representing whether vertex *i* is in the solution hitting set. There are also |Σ|^*k*^ variables *L*_*i*_ representing an upper bound on the number of edges in the longest path ending at vertex *i*. The constraints on *L*_*i*_ guarantee that the vertices chosen remove all *ℓ*-long paths (*ℓ* = *L* − *k*) from the graph. The ILP is defined as follows:

$$\begin{array}{lll} \text{minimize:} & \sum_{i=1}^{|\Sigma {|}^{k}}x_{i}, & \\ \text{subject}\;\text{to:} & x_{i}\in \left\{0,1\right\}, & 1\leq i\leq |\Sigma {|}^{k}\\ & 0\leq L_{i}\leq l-1, & 1\leq i\leq |\Sigma {|}^{k} \end{array}$$ (8)

$$L_{v}\geq 1+L_{u}-lx_{v},\;(u,v)\in E$$ (9)

Here *E* contains all |Σ|^*k*+1^ possible edges. The constraint on edge (*u*, *v*) requires that if *v* is not in the set then *L*_*v*_ ≥ 1 + *L*_*u*_. The validity of this formulation is proven in the Appendix (see Appendix, Subsection Validity of the ILP formulation in S1 Text).

The number of variables and constraints grows exponentially in *k*, making it hard to use for *k* > 7. However, the ILP solver can start from a feasible solution produced by one of the DOCKS algorithms and improve that solution for a limited set time.

### Handling larger *k*

The DOCKS variants described above have exponential dependence in *k* in both runtime and memory usage. Hence, the range of *k* values to which they can be applied is limited. To extend this range, we present below a procedure to construct a universal *k*-mer hitting set by extending UHSs computed for smaller *k* values. Given a set *U*_*K*, *L*_ and integer *j*, we can construct set *U*_*k*+*j*, *L*+*j*_ by concatenating all possible *j*-mers over Σ to each *k*-mer in *U*_*k*, *L*_. Formally,

$$U_{k+j,L+j}=\left\{w\cdot x|w\in U_{k,L},x\in \Sigma^{j}\right\}$$ (10)

To see that *U*_*k*+*j*, *L*+*j*_ is a universal (*k* + *j*)-mer hitting set, denote by *S* an (*L* + *j*)-long sequence. By definition, there must be at least one *k*-mer *w* ∈ *U*_*k*, *L*_ that hits *S*’s *L*-long prefix. *U*_*k*+*j*, *L*+*j*_ contains all (*k* + *j*)-mers *w* ⋅ *x*, where *x* is any *j*-mer. Thus, it must contain a (*k* + *j*)-mer that hits *S*.

For example, by appending all possible 10-mers to each 10-mer in *U*_10,20_ we obtain *U*_20,30_. The size of the set *U*_10,20_ is |*U*_10,20_| = *c* ⋅ *dec*_10_, where ${dec}_{10}\approx \frac{4^{10}}{10}$ is the size of a minimum decycling set for *k* = 10 (Eq 11). Here *c* ≥ 1 is the approximation factor obtained by the UHS. Then, the size of *U*_20,30_ is $|U_{20,30}\left|=\right|U_{10,20}|\cdot 4^{10}=c\cdot {dec}_{10}\cdot 4^{10}\approx c\cdot \frac{4^{20}}{10}=2c\cdot \frac{4^{20}}{20}$ by this construction. This is approximately |*U*_20,30_| ≈ 2*c* ⋅ *dec*_20_, i.e. the approximation factor doubled.

## Results

### A theoretical lower bound for |*U*_*k*, *L*_|

For a given *k*-mer *w*, its *conjugacy class* is the set of *k*-mers obtained by rotation of *w*. Conjugacy classes form cycles in the de Bruijn graph and form a partition of the *k*-mers. The number of conjugacy classes over all *k*-mers is given by [16]:

$$C\left(|\Sigma |,k\right)=\sum_{i=1}^{k}{\left|\Sigma \right|}^{\gcd(i,k)}/k.$$ (11)

A decycling set necessarily contains a *k*-mer from each conjugacy class. Golomb’s conjecture, proved by Mykkeltveit [17], states that the smallest decycling set has cardinality *C*(|Σ|, *k*). Consequently, a minimum hitting set *U*_*k*, *L*_ has a size ≥ *C*(|Σ|, *k*) ≥ |Σ|^*k*^/*k*.

Table 1 reports *L*_*max*_, the length of the longest sequence in a complete de Bruijn graph after a minimum decycling set computed using Mykkeltveit’s algorithm is removed, for *k* = 2 to 14. For this range of *k*, the length of sequences avoiding the decycling set can theoretically be appropriate for long-read sequencing technologies, such as PacBio [19] and Nanopore [20], which produce reads of length *L* > 1000. Such long reads are all hit by a decycling set according to Table 1 for *k* ≤ 14 (although a shorter window size may be needed to overcome sequencing errors). However, many short reads can avoid the decycling set. Additional *k*-mers must be selected to obtain a hitting set for shorter sequences. Note that different minimum decycling sets may result in different lengths of the longest path in the remaining DAG. Mykkeltveit’s approach is different from that of Champarnaud et al. (2004), and the former has an advantage in producing solutions with shorter longest paths [16].

**Table 1:** Length of longest sequence avoiding an unavoidable set for different values of *k*. For each value *k*, a minimum decycling set was removed from a complete de Bruijn graph, and the length *L*_*max*_ of the longest sequence, represented as a longest path, was calculated.

*k*	2	3	4	5	6	7	8	9	10	11	12	13	14
*L*_*max*_	5	11	20	45	70	117	148	239	311	413	570	697	931

### DOCKS

We implemented and ran DOCKS over a range of *k* and *L*: 5 ≤ *k* ≤ 10 and 20 ≤ *L* ≤ 200, in increments of 10. The values of *k* are typical lengths for minimizers, and the *L* values are typical lengths of short reads. Note that in some applications, like KMC 2 [8] and Kraken [12], the length of the window used (denoted by *k* there) corresponds to our *L* parameter, and the length of the minimizers (*m* in KMC 2) corresponds to our *k* parameter.

**Fig 1:**
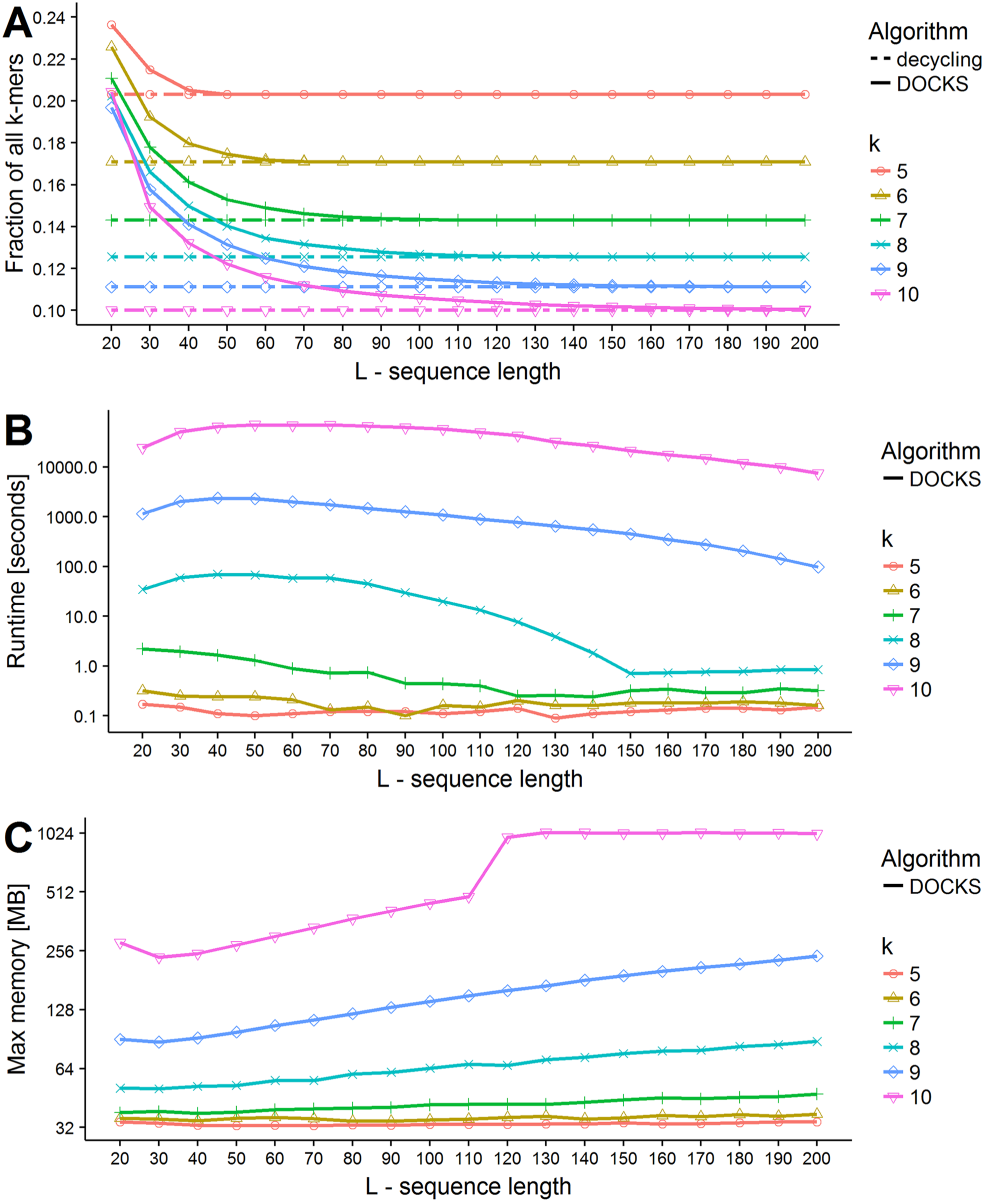
Performance of DOCKS. For different combinations of *k* and *L* we ran DOCKS over the DNA alphabet. (A) Set sizes. The results are shown as a fraction of the total number of *k*-mers |Σ|^*k*^. The broken lines show the decycling set size for each *k*. (B) Running time in seconds. Note that y-axis is in log scale. (C) Maximum memory usage in megabytes. Note that y-axis is in log scale.

The results are summarized in Fig 1. As expected, the fraction of *k*-mers included in the solution set decreases with *L*. It is easier to hit longer sequences as they contain more *k*-mers. In addition, running times and memory usage increase exponentially with *k*. For *k* = 10, DOCKS terminated after more than 2.5 hours and used more than 1 GB of memory. For *k* = 11 and *L* = 20 running time was 128 hours. Hence, DOCKS runtime would be prohibitively long for larger values of *k*. Running times were benchmarked on a single CPU of a 20-CPU Intel Xeon E5-2650 (2.3GHz) machine with 384GB 2133MHz RAM.

Fig 1A also shows the size of the decycling set for each *k*. For *k* = 10 and *L* = 20 the number of added *k*-mers roughly equals the size of the decycling set, while for *k* = 5 and *L* = 20 it is only 20% larger. For all values of *k*, the ratio improves as *L* grows. We also compared DOCKS to a pure greedy algorithm that repeatedly removes a vertex with a maximum hitting number, without removing a decycling set first. For almost all combinations of (*k*, *L*) the size of the produced set, runtime and memory of the greedy algorithm were far greater than those of DOCKS (see Fig A in S1 Text). In particular, the greedy algorithm’s runtime was greater by a factor of more than 1000 for *k* = 8 (taking days compared to minutes), and it increased with *L*, as opposed to DOCKS’s runtime, which decreased with *L*.

### DOCKSany

**Fig 2:**
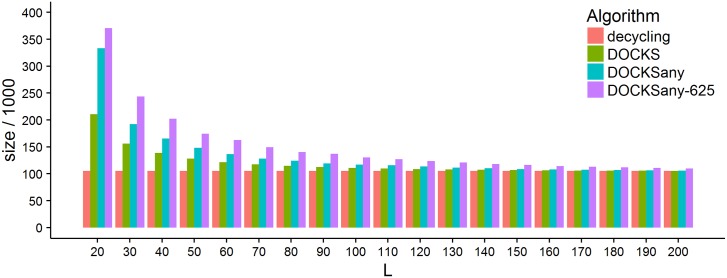
Comparison of the sizes of the universal sets generated by the different heuristics. The histogram shows the size of the universal sets generated by DOCKS, DOCKSany, and DOCKSanyX with *X* = 625. The results are for *k* = 10 and 20 ≤ *L* ≤ 200. The size of the decycling set is provided as a lower bound for comparison.

We ran DOCKSany for 5 ≤ *k* ≤ 11 and 20 ≤ *L* ≤ 200. The results for *k* = 10 are shown in Fig 2 and the full results are in S1 Table and visualized in Fig B in S1 Text. In comparison to DOCKS (see Fig C in S1 Text), the produced sets are larger, especially for smaller values of *L*, and that gap grows with *k*: from 10% for *k* = 5 to 60% larger for *k* = 10. Set sizes of DOCKS and DOCKSany are closer as *L* increases and both approach the size of the decycling set. In terms of running time, on the other hand, we see a great benefit in using DOCKSany as runtimes decrease to a small fraction of the DOCKS running times for the larger values of *k*. We also see reduced memory usage for larger values of *k* and *L* (see the table in S1 Table). Still, DOCKSany becomes impractical for *k* ≥ 13 (runtime for *k* = 12, *L* = 20 was 45 days), so we turn to another heuristic to increase runtime on the expense of larger set sizes.

### DOCKSanyX

We tested the performance of DOCKSanyX for *k* = 10, 20 ≤ *L* ≤ 200 and *X* = 5^*i*^ for 0 ≤ *i* ≤ 5 (Fig D in S1 Text). As expected, the generated set sizes increase with *X*, but the differences are very small for *X* ≤ 125. On the other hand, the running time improves dramatically as *X* increases and the memory usage also improves with *X*, albeit not as dramatically (see S1 Table). Fig 2 compares the sizes of the sets generated by DOCKS, DOCKSany, and DOCKSanyX (for *X* = 625). Remarkably, for *k* = 10, the size of the solution is similar to that of DOCKSany while there is a factor of > 100 × speedup. The results, runtime and memory usage of DOCKSanyX are in S1 Table and visualized in Fig E in S1 Text.

### ILP solutions

**Fig 3:**
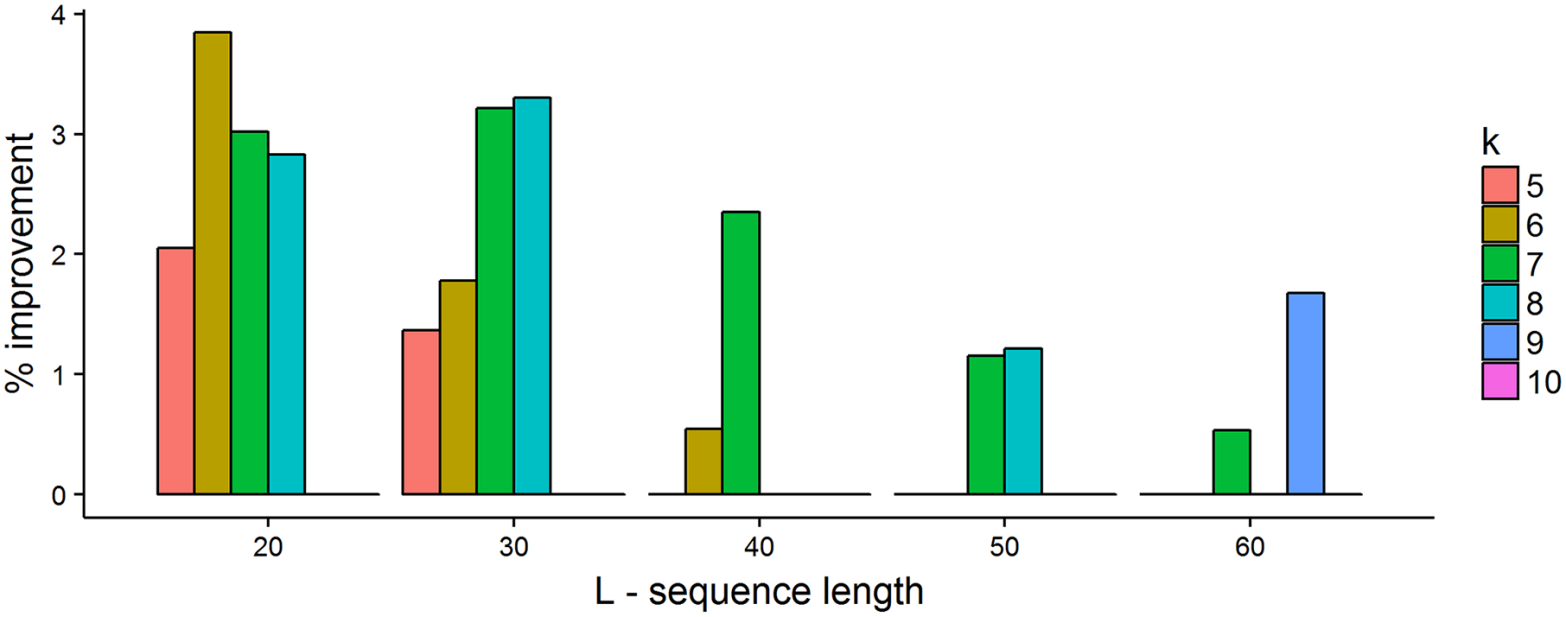
Performance of ILP solver compared to DOCKS. For each combination of 5 ≤ *k* ≤ 10 and 20 ≤ *L* ≤ 200 we ran the ILP solver for up to 24 hours starting from a DOCKS feasible solution. The histograms show the percent improvement of the *k*-mer set size generated by the ILP solver compared to DOCKS. For *L* > 60 and all tested values of *k*, the improvement was <1%.

We solved the ILP using Gurobi 6.5.2 [21] for 5 ≤ *k* ≤ 10 with 20 ≤ *L* ≤ 200. To save time, we set the starting feasible solution to be the DOCKS solution. We let the solver run for up to one day for each *k* and *L*. This did not necessarily produce an optimal solution to the ILP, although the solver was often able to improve on the starting DOCKS solution. In Fig 3, we show the improvement in the solution set size obtained by the ILP over the DOCKS solution. We can see that using the ILP solver leads to minor improvements over the DOCKS solution (0-4%), especially for small *k*. Improvements diminish as *L* increases, since the set sizes approach the theoretical lower bound, i.e., the size of the minimum decycling set. Letting the ILP solver run for longer times may provide further improvements for small values of *L*.

### Comparison to minimizers on several genomes

The minimizer algorithm [1] selects the lexicographically smallest *k*-mer in each window of *w* consecutive *k*-mers in order to reduce storage size for sequence comparison. We can improve the minimizers algorithm by choosing the lexicographically smallest *k*-mer that is in the DOCKS set for the corresponding *k* and *L* parameters (i.e. *L* = *k* + *w* − 1). Such a *k*-mer is guaranteed to exist, as by construction, every window of length *w* contains a *k*-mer in the UHS. We ran the minimizer selection algorithm and DOCKS-based selection on four different genomes, using *k* = 10 and *L* = 30: the entire human reference genome (GRCh38), the bacteria *A. tropicalis* strain NBRC 16470, and *C. crescentus* strain CB15, the worm C. elegans assembly WBcel235. For comparison, we also included the results when using the minimizer according to a random ordering of the *k*-mers, instead of lexicographic. This random ordering typically improves over minimizers since it avoids the problem of always selecting the common poly-A homopolymer.

Table 2 shows that DOCKS selects far fewer *k*-mers and those *k*-mers are more widely spread apart in the sequence. The advantage of DOCKS grows as the sequence length increases, having a size ≈ 85% of the next-best method for the small bacterial genome, ≈ 50% for the larger *C. elegans* genome, and only ≈ 40% for the human genome.

**Table 2:** The number of 10-mers needed to hit all 30-long sequences in four genomes: Two bacterial genomes *A. tropicalis*, *C. crescentus*, the worm *C. elegans* and a mammal genome, *H. sapiens*. The genome sizes are quoted after removing all *N*s and ambiguous codes. We tested three algorithms: minimizers picking the lexicographically smallest 10-mer, minimizer picking the first in a random *k*-mer ordering, and selection using the set produced by DOCKS. In case of multiple DOCKS-selected 10-mers in the 30-long window, the lexicographically smallest was chosen. *# mers* is the number of distinct 10-mers selected, and *avg. dist.* is the average distance between two selected 10-mers.

Species	Genome size (Mbp)	Method	# mers (thousands)	avg. dist.
*A. tropicalis*	0.393	lexicographic	32.9	9.48
		randomized	28.0	11.0
		DOCKS	23.7	12.4
*C. crescentus*	4	lexicographic	114.0	10.2
		randomized	89.6	11.0
		DOCKS	66.0	12.4
*C. elegans*	100	lexicographic	286.0	8.83
		randomized	277.0	11.0
		DOCKS	145.0	12.4
*H. sapiens*	2900	lexicographic	543.0	9.13
		randomized	389.0	10.9
		DOCKS	154.0	12.1

## Discussion

We presented the DOCKS algorithm, which generates a compact set of *k*-mers that together hit all *L*-long DNA sequences. Such compact sets have many applications in sequence analysis, including space efficient data structures and large-scale sequence analysis. We tested the sets produced by our algorithm in an application that requires finding a small set of 10-mers hitting all 30-long words in the input genomes. Compared to minimizers, the current state of the art, our sets were almost 2.5-3.5 times smaller for the human genome. We could produce sets for the range of *k* = 5 to 10 and *L* = 20 to 200, and the results show that for *L* > 100 the size of the solution is quite close to the theoretical lower bound. We expect the sets produced by our approach to be useful and improving a variety of biological applications that require complex analysis of numerous sequences.

We see the benefit of our compact UHSs in many data structures and algorithms that analyze high-throughput sequencing data. For example, we expect that binning-based *k*-mer counting applications, such as KMC 2 [8], can reduce the number of bins, and thus the number of disk accesses, using universal *k*-mer hitting sets. Analyses that rely on *k*-mer counting, such as metagenomic binning as implemented in Kraken [12], will also see improved computational resource usage. The minimizer idea has been widely deployed, and universal hitting *k*-mers can typically be used as a drop-in replacement, improving computational performance.

The good performance of the algorithms can be attributed to their two phase approach. In the first phase we optimally and rapidly remove a minimum-size set that hits all infinite sequences, which also takes care of many *L*-long sequences. In the second phase we greedily remove *k*-mers that hit remaining *L*-long sequences. Overall efficiency is primarily due to the first phase, which runs in time *O*(*k*) times the size of the output. In the second phase dynamic programming is used, providing running time polynomial in the output size.

We developed two additional variants of DOCKS that reduce the runtime and memory usage at the price of increasing the size of the set created. DOCKS can provide a solution for *k* = 10, DOCKSany for *k* = 11, and the fastest variant, DOCKSanyX for *k* = 13 (with *X* = 10000) with *L* = 200, within a day. Note that all heuristics are bound to hit a limit since their runtime depends exponentially on *k*. This is an inherent property of the problem and its output size. Still, we manage to increase *k* by one or two using each heuristic. In partial remedy, we also proposed a construction that can push that limit further at the expense of solution size.

Our approaches are heuristic in nature. This is not surprising, since as we show, the problem of finding a minimum (*k*, *L*)-hitting set for a given set of sequences is NP-hard. Moreover, even after removing an optimal decycling set, one needs to solve the problem of finding a minimum vertex set that hits all *L*-long sequences in a directed acyclic graph, which is NP-hard. Hence, DOCKS usually produces sub-optimal solutions. For example, for *k* = 4 and *L* = 10 the optimal solution obtained by solving an ILP formulation had size 89, compared to 91 produced by DOCKS. In fact, our tests show that if further reduction to the hitting set size is needed, starting from the DOCKS solution and improving it using ILP is a good strategy, at least for small values of *k*.

Our study raises several open problems. First, is there a characterization for a minimum universal (*k*, *L*)-hitting set similar to the characterization of decycling sets by Mykkeltveit [17]? That is, does there exist an algorithm polynomial in *k* and *L* that can check if a *k*-mer belongs to a particular universal (*k*, *L*)-hitting set. The fact that MINIMUM (*k*, *L*)-HITTING SET on a given set of input sequences is NP-hard still leaves the universal case open. A related question is whether one can find an algorithm that generates an optimal (universal) (*k*, *L*)-hitting set while requiring work polynomial in the output set size. This is particularly interesting for the universal case, where the input is only the values *k* and *L* and the output size is > |Σ|^*k*^/*k*. Second, is the problem of minimum *ℓ*-path cover in a DAG *G* polynomial when *G* is a subgraph of a de Bruijn graph? We know it is hard for a general DAG, but the specific structure of de Bruijn graphs may make the problem easier. Third, the bottleneck to DOCKS running time is the second phase, which currently re-calculates the vertex hitting numbers on each iteration. Can one find a dynamic algorithm that updates these numbers more efficiently after the removal of one vertex? Fourth, is there a tight upper bound on the number *p* of vertices that will be removed by the greedy heuristic? Fifth, can we give an upper bound or a tighter lower bound on the size of *U*_*k*, *L*_?

### Conclusion

We demonstrated the ability of DOCKS to generate compact sets of *k*-mers that hit all *L*-long sequences. These *k*-mer sets can be generated once for any desired value of *k* ≤ 13 and *L* and then readily used for many different purposes. For example, we produced a set of only 700 6-mers out of a total of 4096 that hits every sequence longer than 70 bases—a typical read length for many sequencing experiments—enabling efficient binning of reads. Our compact sets can improve many of the applications that currently use minimizers, as we showed that they are both smaller and more sparsely distributed across genome sequences.

## Supporting information

S1 Table Set size, running time and memory usage of DOCKS, DOCKSany, DOCKSanyX, and the greedy algorithm for the hitting set problem. The table contains solution set size, time in seconds and memory in KB for DOCKS, DOCKSany, DOCKSanyX and the greedy approach algorithms. Note that the reported times are for individual runs of each (*k*, *L*) pair, but the sets for all longer *L* values are computed when computing the (*k*, *L* = 20) set with DOCKSany or DOCKSanyX and the runtime can be amortized across all of these calculations.
(XLSX)

S1 Text Supplementary figures and theoretical proofs. (PDF)
