# Supplementary material for: Designing small universal k-mer hitting sets for improved analysis of high-throughput sequencing
Source: PLoS Comput Biol. 2017 Oct 2;13(10):e1005777. doi: 10.1371/journal.pcbi.1005777 (PMC5645146; doi:10.1371/journal.pcbi.1005777)
Supplement: S1 Text — (PDF) [file pcbi.1005777.s002.pdf]

Supporting Information

Fig A

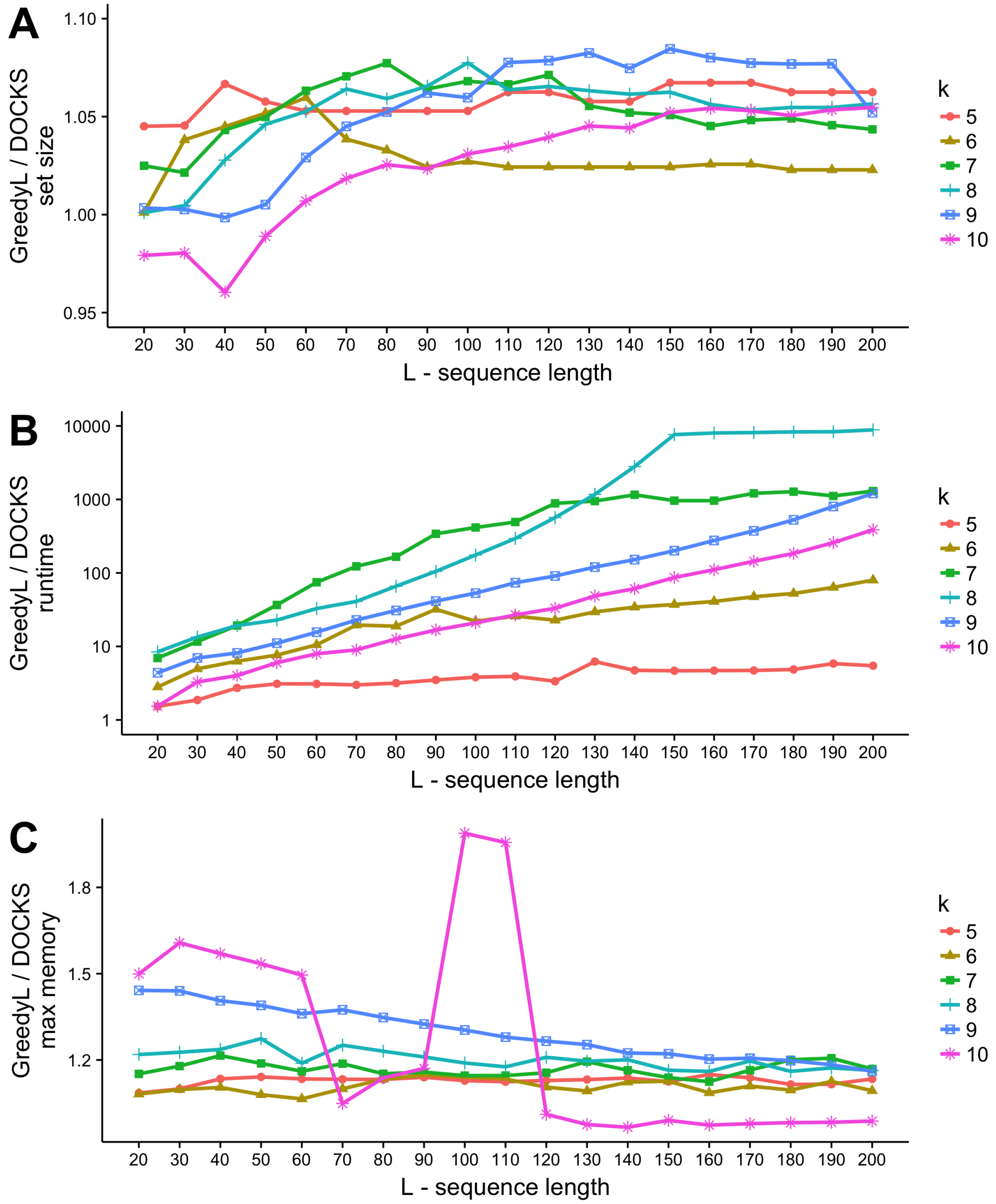

Performance of the greedy algorithm compared to DOCKS. The graphs show the ratio between the greedy algorithm and DOCKS in terms of (A) the k-mer set size generated; (B) the runtime used; (C) the max memory used.

Fig B

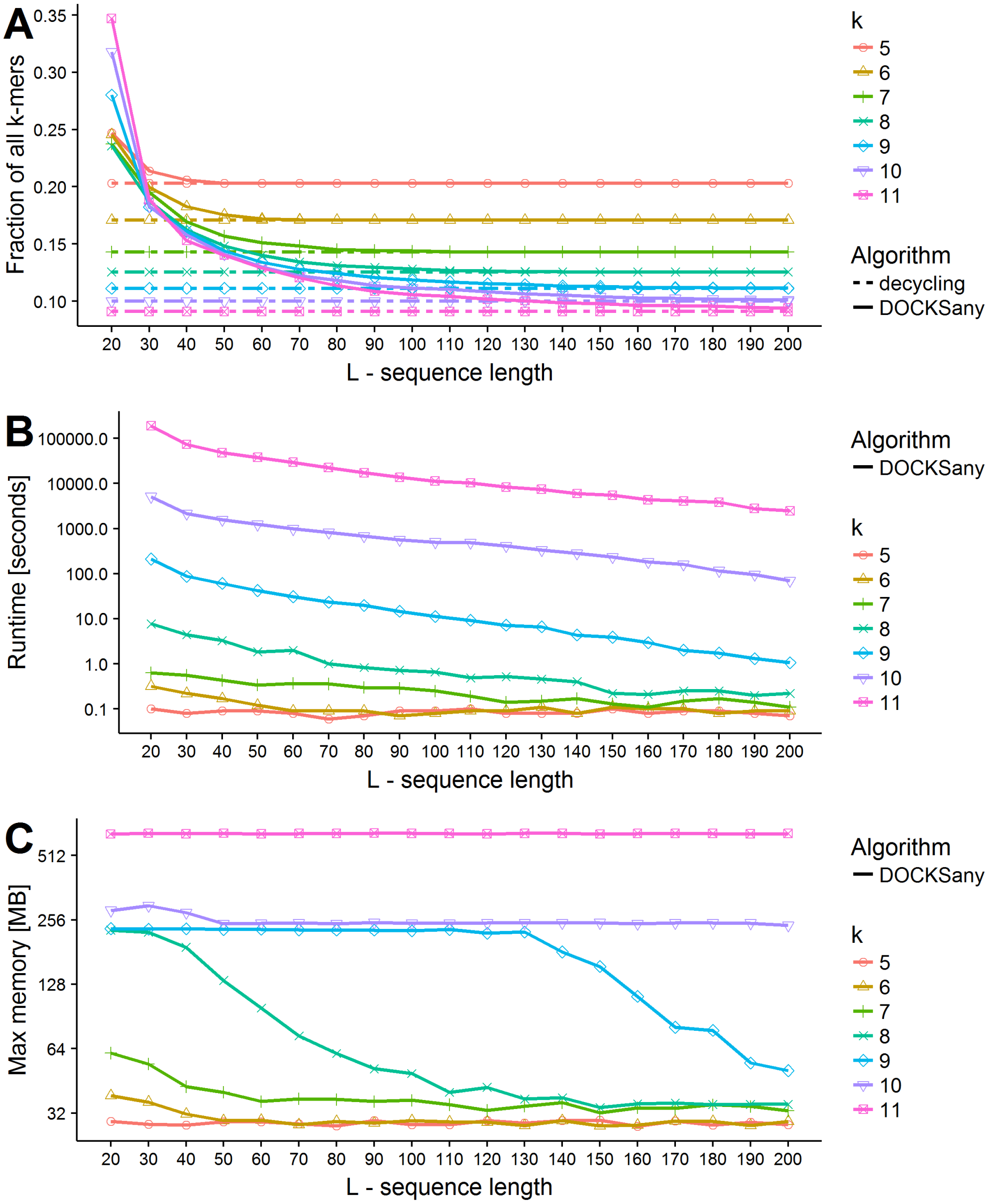

Performance of DOCKSany. For different combinations of k and L we ran DOCKSany over the DNA alphabet. (A) Set sizes. The results are shown as a fraction of the total number of k-mers $|\Sigma|^k$. The broken lines show the decycling set size for each k. (B) Running time in seconds. Note that y-axis is in log scale. (C) Maximum memory usage in megabytes. Note that y-axis is in log scale.

Fig C

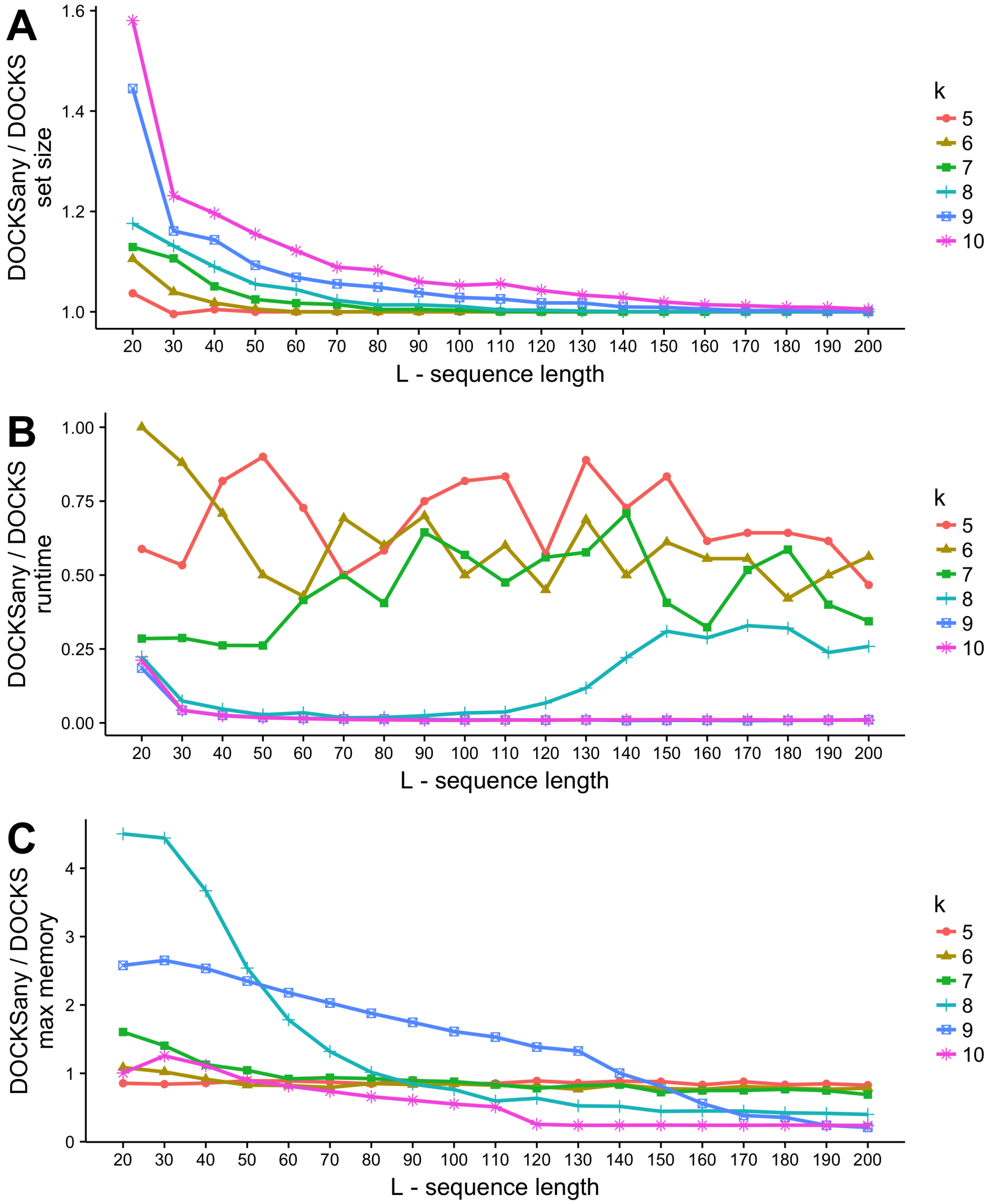

Performance of DOCKSany compared to DOCKS. The graphs show the ratio between DOCKSany and DOCKS for (A) the k-mer set size generated; (B) the runtime used; (C) the maximum memory used.

Fig D

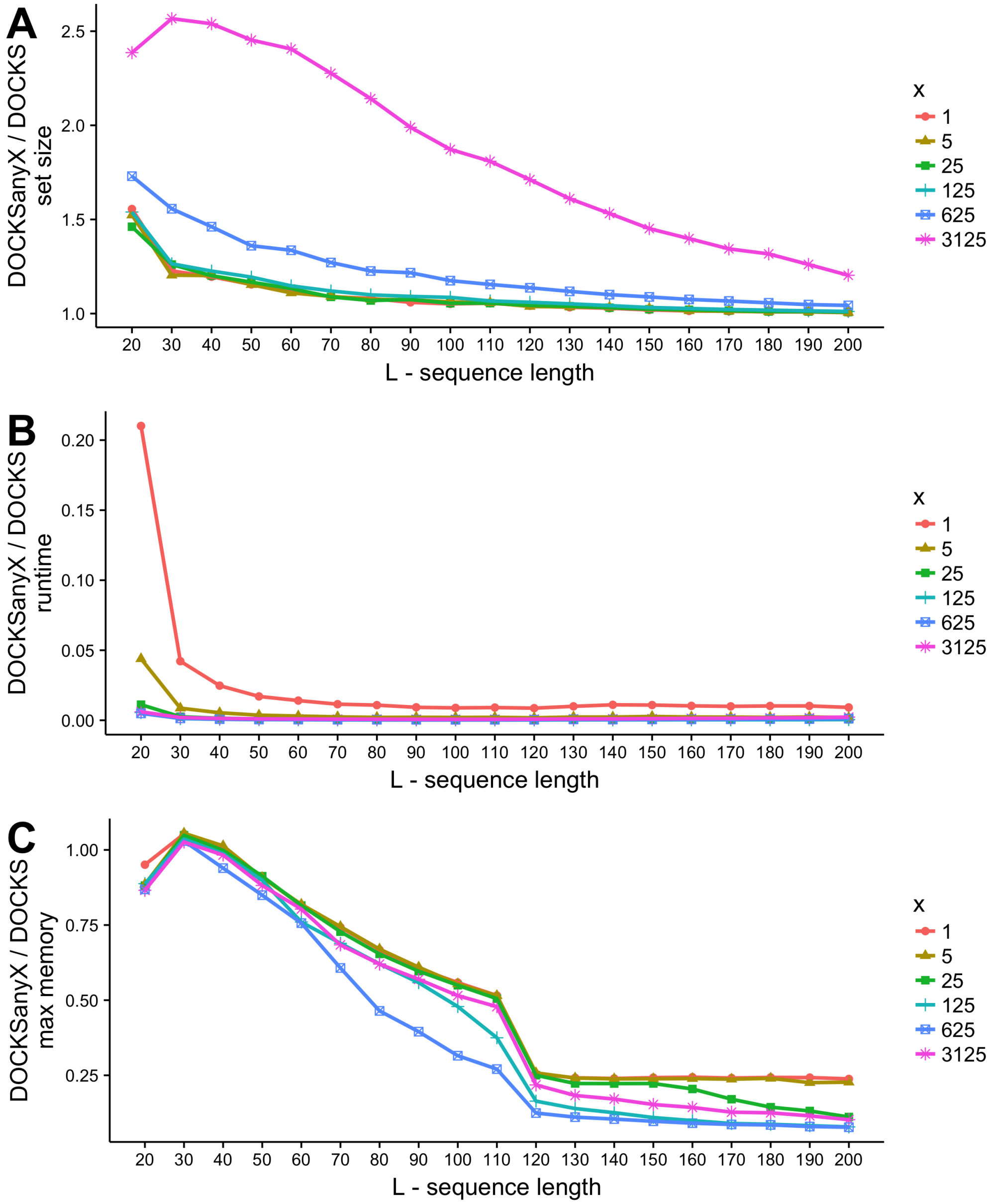

Performance of DOCKSanyX. The graphs show the ratio between DOCKSanyX and DOCKS on $k = 10$ for (A) the k-mer set size generated; (B) the runtime used; (C) the maximum memory used.

Fig E

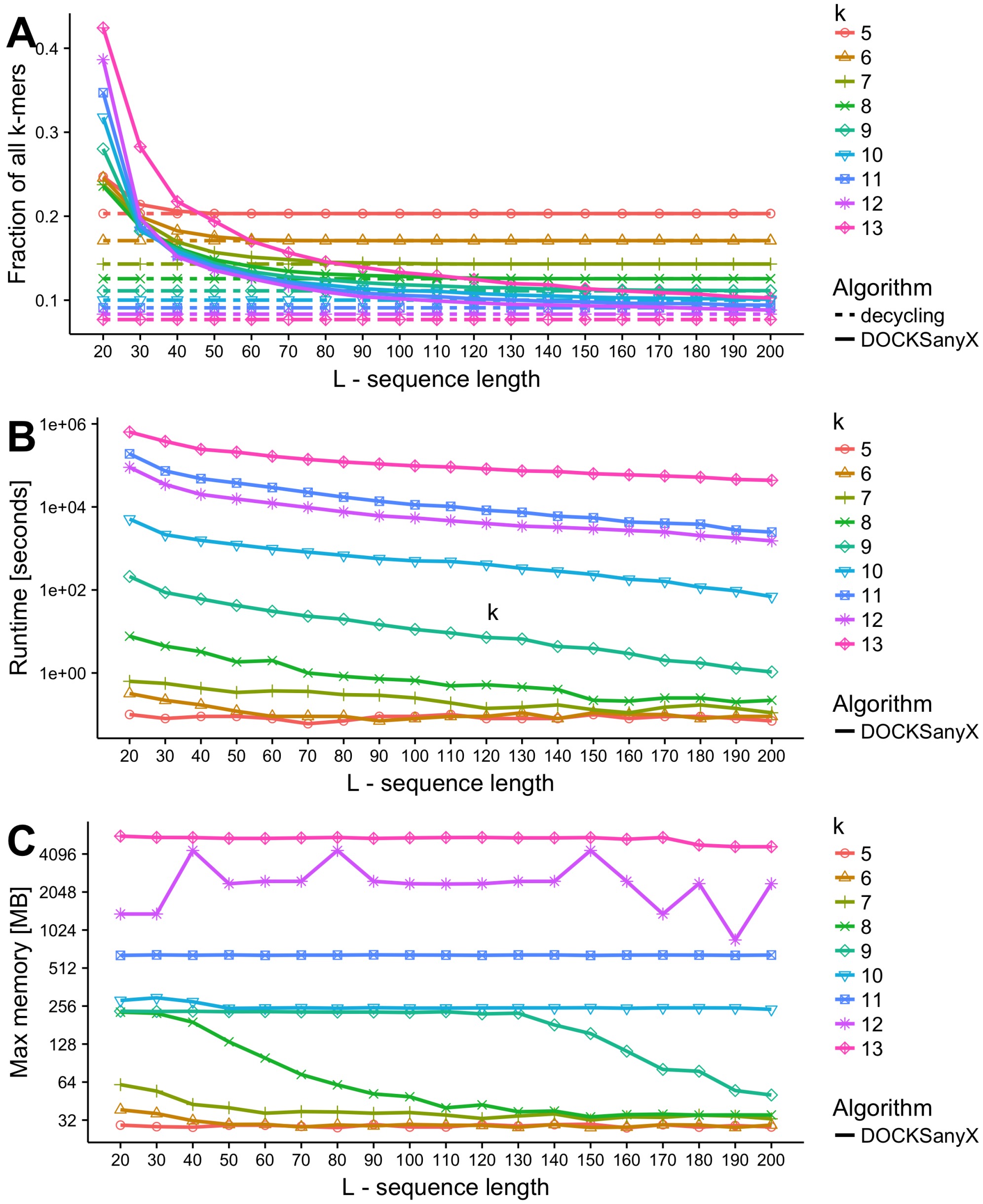

Performance of DOCKSanyX. For different combinations of k and L we ran DOCKSanyX over the DNA alphabet. X value was 1 for $5 \leq k \leq 11$, 100 for $k = 12$ and 10000 for $k = 13$. (A) Set sizes. The results are shown as a fraction of the total number of k-mers $|\Sigma|^k$. The broken lines show the decycling set size for each k. (B) Running time in seconds. Note that y-axis is in log scale. (C) Maximum memory usage in megabytes. Note that y-axis is in log scale.

Appendix

In this section we prove theoretical results used in the body of the paper.

NP-hardness of MINIMUM (k, L)-HITTING SET

One of the motivations for a universal k-mer set comes from the fact that the problem of finding a minimum-size k-mer set that hits every string in a given set of L-long strings is NP-hard. The hitting set problem, if a given set of target sequences is part of the input, is as follows:

MINIMUM (k, L)-HITTING SET

INSTANCE: Set S of L-long sequences over Σ and k.

VALID SOLUTION: Set X of k-mers s.t. $S \subseteq hit(X, L)$.

GOAL: Minimize $|X|$.

We prove that MINIMUM (k, L)-HITTING SET is NP-hard. For simplicity, we study the problem on the DNA alphabet, but it can be easily generalized to any finite alphabet Σ. We show a reduction from HITTING SET [1]. While the problems look similar, HITTING SET is a more general case than our problem, since in HITTING SET the subsets are arbitrary, while in MINIMUM (k, L)-HITTING SET problem each subset is made of overlapping k-mers. Hence, the hardness of the former does not directly imply hardness of the latter.

Theorem 1. *MINIMUM (k, L)-HITTING SET is NP-hard.*

Proof. Given an input to HITTING SET, a set S of subsets of $E = \{e_1 \dots e_n\}$, we generate an input to MINIMUM (k, L)-HITTING SET problem as follows: Denote by m the size of the maximum cardinality set, i.e. $m = \max_{S_i \in S} |S_i|$. We choose $\ell = \lceil \log_2(\max(m, n)) \rceil$, $L = 3\ell m$ and $k = 2\ell$. We map each set $S_i \in S$ to a k-long binary representation of i, where instead of bits we use nucleotides `C` and `G`. We map each element $e_j \in E$ to a k-long binary representation of j, where instead of bits we use nucleotides `A` and `T`. We call these representations the set's $\{C, G\}$-representation and the element's $\{A, T\}$-representation and denote them by $f_{CG}(S_i)$ and $f_{AT}(e_j)$.

We generate a sequence set T, which is the input to MINIMUM (k, L)-HITTING SET. For each set $S_i \in S$ we generate a sequence that contains all of its elements' $\{A, T\}$-representations, each appearing twice consecutively and buffered by the set's $\{C, G\}$-representation. Formally, for the set $S_i = \{e_{i_1}, \ldots, e_{i_{|S_i|}}\}$ we create the sequence: $T_i := (\prod_{j=1}^{|S_i|} f_{AT}(e_{i_j}) \cdot f_{AT}(e_{i_j}) \cdot f_{CG}(S_i)) \cdot (f_{AT}(e_{i_1}) \cdot f_{AT}(e_{i_1}) \cdot f_{CG}(S_i))^{m-|S_i|}$ (here $\prod$ indicates concatenation). The new instance T is $\{T_1, \ldots, T_{|S|}\}$.

Denote by T^{OPT} an optimal solution to MINIMUM (k, L)-HITTING SET. If a k-mer contains as a substring a complete $\{A, T\}$-representation w, then the element $f_{AT}^{-1}(w)$ is in the optimal solution to HITTING SET. If a k-mer contains a complete $\{C, G\}$-representation w, then any element from the set $f_{CG}^{-1}(w)$ can be part of the optimal solution. The running time of the reduction is bounded by $O(|S| \times L)$ to generate the input sequence set T. In terms of m and n the running time is $O(|S| \cdot m \cdot (\log(m) + \log(n)))$.

We now prove the correctness of the reduction. We start with proving several properties of the solution.

Lemma 1. *A k-mer that contains a complete $\{A, T\}$-representation w can be replaced by k-mer ww to produce a hitting set of the same cardinality.*

Proof. The k-mer contains a complete $\{A, T\}$-representation w. Thus, it can only hit sequences that contain w. Since the sequences were constructed to contain two adjacent $\{A, T\}$-representations per element, and since this representation is unique, k-mer ww hits the same set of sequences. □

Lemma 2. *A k-mer that contains a complete $\{C, G\}$-representation can be replaced by a k-mer that contains two adjacent occurrences of any $\{A, T\}$-representation from this sequence to produce a hitting set of the same cardinality.*

Proof. A $\{C, G\}$-representation is unique to each sequence. Thus, it can only hit one sequence, and replacing it by any other k-mer from that sequence preserves the hitting properties of the set. □

We now prove the two sides of the reduction:

1. MINIMUM (k, L)-HITTING SET $\Rightarrow$ HITTING SET: all L-long sequences in T are hit by k-mers in T^{OPT}. By Lemmas 1 and 2 we can transform any hitting set

to a hitting set of the same cardinality, but containing only k-mers over $\{A, T\}$. These correspond to elements in an optimal solution of HITTING SET. Assume contrary that there is a smaller solution U to HITTING SET. Then, the set $\{f_{AT}(w) \cdot f_{AT}(w) \mid w \in U\}$ hits all sequences in the k-mer hitting problem, and by that producing a smaller solution, contrary to its optimality.

2. HITTING SET $\Rightarrow$ MINIMUM (k, L)-HITTING SET: denote by S^{OPT} an optimal solution to HITTING SET. Then, a set of k-mers $\{f_{AT}(w) \cdot f_{AT}(w) \mid w \in S^{OPT}\}$ is an optimal solution to MINIMUM (k, L)-HITTING SET. Assume contrary that there is a smaller solution U to MINIMUM (k, L)-HITTING SET. By Lemmas 1 and 2 there is a solution composed of k-mers over $\{A, T\}$. The set of element $\{f_{AT}^{-1}(w_{1:k/2}) \mid w \in U\}$ is a smaller hitting set in HITTING SET, contrary to its optimality.

□

NP-hardness of MINIMUM ℓ-PATH COVER IN A DAG

Our heuristic to find $U_{k,L}$ searches for a minimum ℓ-path cover in the DAG created after removing a decycling set. In the second phase of DOCKS we encounter a special case of the following problem.

MINIMUM ℓ-PATH VERTEX COVER IN A DAG

INSTANCE: A directed acyclic graph $G = (V, E)$ and integer ℓ.

VALID SOLUTION: Vertex set X s.t. $G' = (V \setminus X, E)$ contains no ℓ-long paths.

GOAL: Minimize $|X|$.

This general problem was shown to be NP-hard in [2]. A special case of the problem, for an acyclic subgraph of the de Bruijn graph, arises in the second phase of DOCKS after removing a minimum decycling set. The hardness result motivates the use of heuristics in the second phase.

Validity of the ILP formulation

Lemma 3. *The ILP is a valid formulation of the minimum hitting set problem.*

Proof. Suppose S is a UHS, and define $x_v^* = 1 \iff v \in S$, $L_v^* = 0$ if $v \in S$ and otherwise L_v^* equal to the length of the longest path ending at v. We claim that (x^*, L^*) satisfy the constraints. By construction, (8) holds. To show (9), if $v \in S$ then $0 = L_v^* \geq 1 + L_u^* - \ell$. If $v \notin S$, then $L_v^* \geq 1 + L_u^*$ by the property of the longest path labels. Hence all constraints are satisfied. Conversely, suppose the vectors x^* and L^* solve the ILP. W.l.o.g., we can assume that L^* is integer (otherwise round all coordinates down and all inequalities still hold for the new solution). Define $S = \{i \mid x_i^* = 1\}$. We claim that S is a UHS. Suppose by contradiction there exists a path of ℓ edges $p = (u_0, e_0, u_1, e_1, \ldots, u_\ell)$ in the graph induced by $G_k \setminus S$ (i.e. the DAG induced by removing the set S from the order k de Bruijn graph). Then, $x_{u_i}^* = 0$ for $i = 0, \ldots, \ell$ and summing the inequalities (9) for the edges in the path we get $L_{u_\ell} \geq L_{u_0} + \ell$, which contradicts (8). Hence, S is indeed a UHS. □

References

1. Karp RM. Reducibility among combinatorial problems. In: 50 Years of Integer Programming 1958-2008. Springer; 2010. p. 219–241.

2. Paindavoine M, Vialla B. Minimizing the Number of Bootstrappings in Fully Homomorphic Encryption. In: Revised Selected Papers of the 22Nd International Conference on Selected Areas in Cryptography - SAC 2015 - Volume 9566. New York, NY, USA: Springer-Verlag New York, Inc.; 2016. p. 25–43. Available from: http://dx.doi.org/10.1007/978-3-319-31301-6_2.
